# Serological Evidence of West Nile Virus in Wild Birds in Bangladesh

**DOI:** 10.3390/vetsci7040164

**Published:** 2020-10-28

**Authors:** Ariful Islam, Shariful Islam, Mohammad Enayet Hossain, Jinnat Ferdous, Josefina Abedin, Mohammad Ziaur Rahman, Md. Kaisar Rahman, Md. Ahasanul Hoque, Mohammad Mahmudul Hassan

**Affiliations:** 1 Centre for Integrative Ecology, School of Life and Environmental Sciences, Deakin University, Geelong Campus, VIC 3216, Australia; 2 EcoHealth Alliance, New York, NY 10001-2023, USA; sharifdvm51@gmail.com (S.I.); ferdousjinnat90@gmail.com (J.F.); drjosefinaabedin@gmail.com (J.A.); kaisarrahman@ecohealthalliance.org (M.K.R.); 3 Bangladesh Livestock Research Institute, Savar, Dhaka 1241, Bangladesh; 4 International Centre for Diarrheal Diseases Research, Bangladesh (ICDDR,B), Dhaka 1212, Bangladesh; enayet.hossain@icddrb.org (M.E.H.); mzrahman@icddrb.org (M.Z.R.); 5 Institute of Epidemiology, Disease Control and Research (IEDCR), Dhaka 1212, Bangladesh; 6 Faculty of Veterinary Medicine, Chattogram Veterinary and Animal Sciences University, Chattogram 4225, Bangladesh; md.hoque@my.jcu.edu.au

**Keywords:** seroprevalence, wild birds, c-ELISA, c-PCR, RNA, surveillance

## Abstract

West Nile Virus (WNV) is a vector-borne zoonotic disease maintained in a sylvatic cycle involving mosquito vectors and birds. To detect WNV and other *flavivirus* infections in wild resident and migratory birds, we tested 184 samples from 19 identified species within nine families collected during 2012–2016 from four districts in Bangladesh. We tested serum samples for the immunoglobulin G (IgG) antibody against WNV using competitive Enzyme-Linked Immunosorbent Assay (c-ELISA), whereas tracheal and cloacal swabs were subjected to consensus Polymerase Chain Reaction (c-PCR) for the detection of the flavivirus RNA. Overall, we detected 11.9% (*n* = 22; 95% CI: 0.07–0.16) samples were seropositive, including 15.9% in the migratory wild birds and 10.7% in the resident wild birds. The migratory wild Tufted duck showed 28.5% seropositivity, whereas the resident wild house crows showed 12.5% seropositivity. None of the swab samples was positive for flavivirus RNA infection (0%, *n* = 184; 95% CI: 0–0.019). These study findings recommend continued surveillance for early detection and to better understand the epidemiology of WNV and other flavivirus circulation in both birds and mosquitoes in Bangladesh.

## 1. Introduction

West Nile Virus (WNV) is an emerging zoonotic arbovirus, having enveloped positive-sense single-stranded RNA belonging to the family *Flaviviridae* in the genus *Flavivirus* [1]. WNV was first recovered in 1937 from a febrile woman from the West Nile district of Uganda and in birds (crows and columbiformes) in the Nile delta region in 1953 [2]. The detection of WNV in New York in the US in 1999 showed the first introduction of an Old World flavivirus into the New World [3], and within three years the virus had spread throughout the US, moving through Canada, Mexico, Central America, and the Caribbean, and down to Argentina by 2006 [4]. WNV is now considered an endemic disease in the US. In the 77 years since its detection, the virus has spread worldwide on all continents except for Antarctica and caused epidemic outbreaks, and is now considered the most critical causative agent of viral encephalitis globally [5]. WNV naturally maintains a bird–mosquito–bird cycle, but human and other mammalian infections have been reported worldwide, including Asia. Humans and horses are infected as dead-end hosts, and a variety of *Culex* spp. mosquitoes are competent vectors of the virus [6]. The virus causes infrequent febrile illness; nervous disorder (encephalitis); and mortality to humans, horses, and wild birds [7]. In the tropical region, members of *Culex* spp. and *Aedes* spp. are mainly responsible for the transmission of the virus [8]. Depending on the season, migratory birds annually overfly countries in both the Northern and Southern hemisphere [9], thus playing an essential role in the spread of WNV in Asia, Africa, and Europe [1]. When migratory birds visit different tropical and subtropical countries to avoid the intense cold weather during winter, they carry viruses, including avian influenza [10,11,12,13] and others, from temperate to tropical regions. The presence of WNV has been reported in different countries, like Mexico, the USA, India, Pakistan, and Germany, covering various continents, including Asia, Europe, Australasia, and Africa. Bangladesh lies in the subtropical region and has a moderately warm temperature [14]. These climatic conditions may increase WNV transmission by decreasing the duration for vector development and increasing the rate of mosquito biting and viral replication [15]. The virus is widespread in humans and birds of Asian countries. In South Korea, it has been reported that 0.3% of migratory wild birds had immunoglobulin against WNV [16]. WNV seroprevalence was reported as being 11.6% [17] to 14.5% [8] in humans and 1.6% in wild residents and migratory birds [18] in India. Moreover, WNV was detected in humans and horses in Pakistan [19,20]. Recently, WNV has been identified in a human patient at the International Center for Diarrheal Disease Research, Bangladesh (ICDDR,B) (report from Prothomalo, 25 September 2019). To the best of our knowledge, no study has been conducted yet in Bangladesh to determine the status of WNV in animals, especially resident and migratory wild birds. Therefore, the present study aimed to detect evidence of WNV in resident and migratory wild birds of Bangladesh, as the baseline information for future research.

## 2. Materials and Methods

### 2.1. Study Time and Location

**Figure 1:**
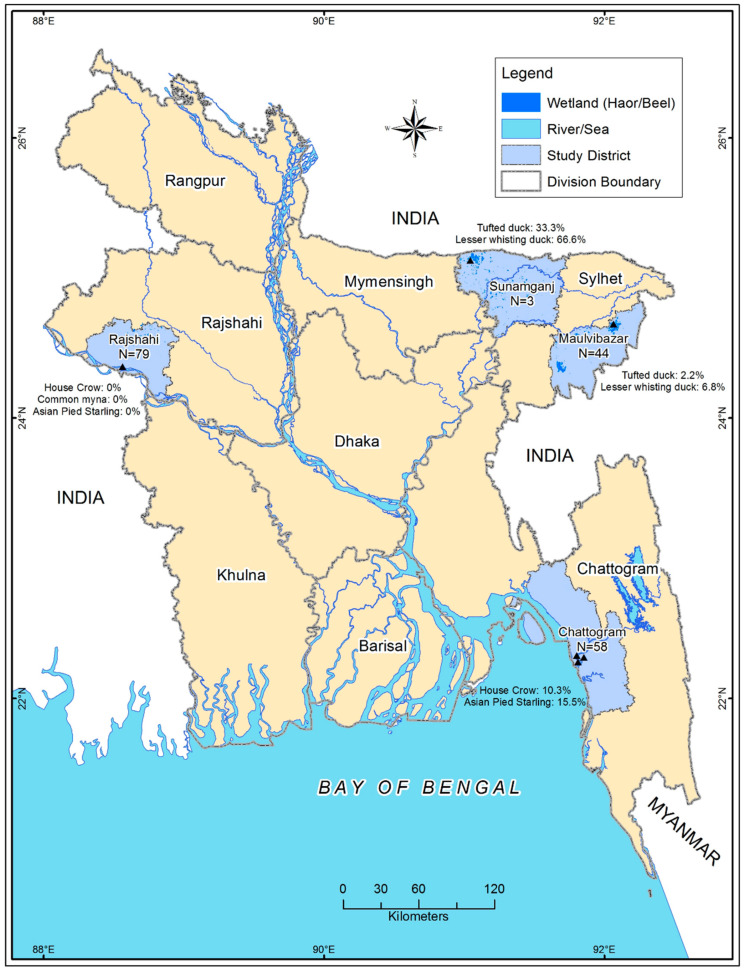
Map of Bangladesh. Sampling locations plotted using the spatial analyst tool of ArcGIS (ArcMap, version 10.2, Environmental Systems Research Institute, Redlands, CA, USA). Study sites for estimating seroprevalence of West Nile Virus in resident and migratory wild birds (*n* = 184) with their sample size and % of positive species in four different locations of Bangladesh from December 2012 to February 2016.

We conducted a cross-sectional study to screen WNV from swabs and serum samples of 12 species of resident and migratory wild birds (*n* = 184) from December 2012 to February 2016 at (Supplementary Table S1) four different sites (Chattogram, Sunamgonj, Moulavibazar, and Rajshahi district) of Bangladesh (Figure 1). We selected the study sites based on the migratory bird population. Chattogram and Rajshahi are located on the bank of River Padma and Karnaphuli, respectively, where different migratory birds visit during winter (November to February) [21]. In Sunamgonj and Moulavibazar, we collected samples from Tanguar and Hakaluki Haor (major wetland of Bangladesh), as a popular hub for resident as well as migratory wild birds [22].

### 2.2. Sample Collection and Laboratory Analysis

We used mist nets and leg nooses to capture the wild birds [11,23]. All trapped resident and migratory birds were sampled humanely. We collected blood (0.5–3.0 mL, in all cases < 1% of body weight) samples by venipuncture aseptically from jugular or wing or leg vein and then immediately placed them into 3 mL serum tubes with serum activator (Vacutainer) with unique identity numbers. Blood samples tubes were subsequently allowed to clot on ice packs in a cool box, followed by centrifugation at 10,000 rpm for 30 min. We separated serum within six hours of blood collection, placed in a cryovial (Corning). The serum samples were stored in liquid nitrogen dewar (Princeton Cryogenics) in the field and then transferred to a −80 °C freezer in the laboratory.

Serum samples were then transferred into cryovials and preserved at −80 °C [24]. We evaluated the serum samples for WNV-specific antibody using competitive Enzyme-Linked Immunosorbent Assay (c-ELISA) (ID Screen^©^ West Nile Competition, IDVet, Montpellier, France) following the protocol described by [25]. The c-ELISA kit was designed to detect IgG antibodies from multiple host species, such as birds and horses, against the envelope protein (prE) of WNV. The c-ELISA is highly sensitive and specific >95%, and the freeze-dried positive horse serum was used as internal reference material for quality control [26]. The plates were pre-coated by the manufacturer, and the c-ELISA made use of a direct format that uses a monoclonal anti- prE HRP (competition) antibody. We interpreted the tested serum samples as positive when the residual binding ratios (S/N percentage, OD sample divided by OD negative control) were equal to or lower than 40% as recommended by the manufacturer [27]. The Optical density (OD) of each well was read using an ELISA reader at a wavelength of 450 nm.

We also collected cloacal and oropharyngeal swabs using sterile polyester swabs with plastic shafts (Fisher) along with blood samples, from each bird. Swab samples were obtained from birds by inserting swab sticks into the vent (until fecal contamination) for cloacal swabs and oropharyngeal airway and wall of oropharynx for oropharyngeal swabs. Each of the cloacal and oropharyngeal swab samples was placed independently into a cryovial containing one mL of sterile viral transport media [28]. We tested swab samples using c-PCR, according to a published protocol [29] targeting the highly conserved flavivirus NS_5_ gene, which contains short amino acid motifs that are 100% identical in all known flaviviruses. We used the universal control plasmid (synthetic gene) as a positive control in consensus PCR, and the sequence included in the universal control for this flavivirus assay is WNV, as described by [30,31]. The universal positive control was created to run positive control material for the c-PCR screening. These structures are made up of sequential (non-overlapping) primer-binding sites for all assays, interspersed with short stretches of synthetic sequence. The positive control allows for the universal amplification of sequences from viruses within a given family or genus, and the subsequent discernment of viral strains within to confirm the successful execution of the assay. The assays also verify the recognition of sample contamination, given that amplified products contain a series of primer-binding sites rather than a real viral sequence [31].

### 2.3. Statistical Analysis

We used Microsoft Office Excel 2013 for data management and STATA/IC- 13 (StataCorp, 4905, Lake Way Drive, College Station, TX 77845, USA) for performing data analysis. We analyzed the collected data (both demographic and laboratory) and expressed the results as frequency (*n*), percentage (%), and 95% confidence interval (CI). Fisher’s exact test was done to compare the variables and their significance. The *p* value < 0.05 was considered a statistically significant difference.

### 2.4. Ethical Approval

We captured captive wild birds using the approval of the Bangladesh Forest Department, The Peoples Republic of Bangladesh (permit reference number: WASU/FAO/PSWMID-6/2012/58; Date: 23 July 2013). Handling and sampling of birds were approved by the Chattogram Veterinary and Animal Sciences University Animal Experimentation Ethics Committee (permit ref. no. CVASU/Dir (R and E) AEEC/2015/02), Bangladesh. Birds were released without injury or harm into their cages after sampling, and all efforts were made to minimize animal suffering throughout our research.

## 3. Results and Discussion

The overall sero- and viral prevalence of WNV in wild birds of Bangladesh was 11.9% (*n* = 184; 95% CI: 0.07–0.16) and 0% (*n* = 184, 95%CI: 0–0.019), respectively. This result was higher than the findings of a study conducted in Spain (1.96%) using the ELISA test [6] and lower than another study of Portugal (19.8%) [32]. The hot and humid environment of Bangladesh can increase the spreading and propagation of the arthropod-borne virus [33]. The highest seroprevalence was found in wild birds from Chattogram (25.8%). However, no samples from Rajshahi were positive either in c-ELISA or in c-PCR. One study from India also reported a negative result of WNV in RT-PCR. However, they found antibodies in the same samples [18]. Again, the seroprevalence was higher in the birds captured from the wetland (14.8%) than plain land (10.9%) (Table 1). Wetlands are a suitable place where domestic birds (especially domestic ducks) and resident wild birds meet with migratory birds in winter. Scientists from Japan and South Korea have recently found evidence of WNV in domestic ducks that had contact with migratory birds in wetlands [16,34]. Additionally, a large population of mosquito in the wetland, along with low human population density, can help to maintain the virus circulation in the birds of the sampled area [6].

The WNV seroprevalence in the study was highest in tufted duck (28.5%), followed by lesser whistling duck (22.7%), Asian pied starling (20.9%), and crow (12.5%). Contrarily, none of the northern pintail, white-throated kingfisher, rock pigeon, seagull, house sparrow, common moorhen, common myna, and barn owl were positive for WNV (Table 1). We sampled all the tufted and lesser whistling ducks from Hakaluki Haor, and Moulavibazar, the most significant wetland of Bangladesh. This area also serves as the primary wintering ground for birds migrating in both the Central Asian and Eastern Asian–Australian flyways [35]. The domestic ducks and resident wild birds of this area come into contact with migratory birds; this was considered as the first-line contact, which may facilitate the dissemination of WNV to other species. Previously, different bird species like hummingbird, cormorant, ring-billed gull, mourning dove from Mexico [4], northern cardinal, rock dove, purple swamphen, little egret, black ibis, spot-billed duck, common coot, mallard, ruff, and purple heron from New York [36] were found seropositive for WNV. However, we found a lower seroprevalence of WNV in house sparrow and chicken than previously reported [36]. Among all songbirds, common grackles had a higher amount of viremia [37]. Moreover, adult birds were found to be more positive than juveniles [38].

The virus is prevalent in birds and humans of countries like India [17,18,39] and Pakistan [19,20]. India shares borders with Bangladesh, which facilitates human and bird movement between the two countries. As a result, WNV can spread to Bangladesh by the movement of humans as well as birds from India. Moreover, climate change and its consequences on the potential vectors of WNV or bird migration routes impact the distribution of the disease [1]. Migratory birds might be an important source of WNV [40] because they come from European countries to Bangladesh every year (as they transit in different countries) to survive from extreme cold, and this may act as a source of transmission to Bangladesh’s resident wild birds. Other factors that influence the viral transmission cycle are season, temperature, humidity, etc. [6,15,41]. Our study had some limitations: we tested a small number of samples and conducted a serological survey without a serum neutralization test (SNT). The SNT is the gold-standard to confirm the positive WNV antibody [42]. Though there is an opportunity of cross-reaction with related viruses, c-ELISA has higher sensitivity and specificity than other types of ELISA [26]. Moreover, several studies from different countries conducted sero survey using c-ELISA and found this test appropriate for WNV in free-ranging and captive birds [43,44,45]. However, we cannot rule out whether the birds were carrying other closely related flaviviruses or not. Usually, the detection of WNV circulation using viral RNA detection is not practical due to the difficulties to find positive samples because of the short period of viremia (usually 4–6 days) caused by infections [7]. Thus, WNV diagnosis and surveillance in hosts, in most cases, relies on serological analysis. Furthermore, WNV IgG-based ELISA is a reliable marker in screening the presence of WNV neutralizing antibodies when vaccination and infection of WNV have been absent in the past. Hence, the usage of the kit fits the purpose of our study [46]. Whilst it is recognized that ELISA tests cannot absolutely define the serological specificity of samples that may contain antigenically cross-reactive epitopes, for example, with closely-related flaviviruses such as Usutu virus or Japanese encephalitis virus [27], this study provides baseline data implying that WNV could be carried by resident and migratory overflying birds.

## 4. Conclusions

The detection of WNV-reactive antibodies for the first time in resident and migratory wild birds of different areas of Bangladesh indicates likely exposure to WNV or other closely related flaviviruses. However, no WNV-genomic RNA was detected, and we, therefore, cannot exclude the possibility that the birds were seropositive for related flaviviruses. Future studies should be directed toward the continuous surveillance of the virus using a serum neutralization test (SNT) in humans and birds to inform and appropriate control measures and enable them to be implemented.

## Figures and Tables

**Table 1:** Univariate association between West Nile Virus seroprevalence and selected factors (N = 184).

Variable	Category	N	Positive *n* (%)	*p* (Fisher’s Exact)
District	Chattogram	58	15 (25.8)	0.00
	Moulavibazar	47	7 (14.8)	
	Rajshahi	79	0 (0)	
Type of birds	Resident wild bird	140	15 (10.7)	0.42
	Migratory wild bird	44	7 (15.9)	
Landscape	Plain	137	15 (10.9)	0.45
	Wetland	47	7 (14.8)	
Family				
Anatidae	Tufted duck	7	2 (28.5)	
	Lesser whistling duck	22	5 (22.7)	0.05
	Northern pintail	12	0	
Sturnidae	Asian pied starling	43	9 (20.9)	
	Common myna	40	0	
Corvidae	House crow	48	6 (12.5)	
Columbidae	Rock pigeon	2	0	
Alcedinidae	White-throated kingfisher	2	0	
Laridae	Seagull (Gangchil)	3	0	
Passeridae	House sparrow	1	0	
Rallidae	Common moorhen	3	0	
Tytonidae	Barn owl	1	0	

## Supplementary Materials

The following are available online at https://www.mdpi.com/2306-7381/7/4/164/s1, Table S1: metadata of resident and migratory wild birds samples collected for West Nile Virus (WNV) surviellance during 2012–2016 from four districts in Bangladesh.
